# Comprehensive analysis of differentially expressed profiles of mRNA, lncRNA, and miRNA of Yili geese ovary at different egg-laying stages

**DOI:** 10.1186/s12864-022-08774-4

**Published:** 2022-08-19

**Authors:** Xiaoyu Zhao, Yingping Wu, Haiying Li, Jiahui Li, Yingying Yao, Yan Cao, Zhiyong Mei

**Affiliations:** College of Animal Science, Xinjiang Agricultural University, Urumqi, 830000 China

**Keywords:** Yili geese, Ovary, Laying period, mRNA, Non-coding RNA, Regulation networks

## Abstract

**Background:**

The development of the ovaries is an important factor that affects egg production performance in geese. Ovarian development is regulated by genes that are expressed dynamically and stage-specifically. The transcriptome profile analysis on ovarian tissues of goose at different egg laying stages could provide an important basis for screening and identifying key genes regulating ovarian development.

**Results:**

In this study, 4 ovary tissues at each breeding period of pre-laying (PP), laying (LP), and ceased-laying period (CP), respectively, with significant morphology difference, were used for RNA extraction and mRNAs, lncRNAs, and miRNAs comparison in Yili geese. CeRNA regulatory network was constructed for key genes screening. A total of 337, 1136, and 525 differentially expressed DE mRNAs, 466, 925, and 742 DE lncRNAs and 258, 1131 and 909 DE miRNAs were identified between PP and LP, between CP and LP, and between CP and PP groups, respectively. Functional enrichment analysis showed that the differentially expressed mRNAs and non-coding RNA target genes were mainly involved in the cell process, cytokine-cytokine receptor interaction, phagosome, calcium signaling pathway, steroid biosynthesis and ECM-receptor interaction. Differential genes and non-coding RNAs, PDGFRB, ERBB4, LHCGR, MSTRG.129094.34, MSTRG.3524.1 and gga-miR-145–5p, related to reproduction and ovarian development were highly enriched. Furthermore, lncRNA-miRNA-mRNA regulatory networks related to ovary development were constructed.

**Conclusions:**

Our study found dramatic transcriptomic differences in ovaries of Yili geese at different egg-laying stages, and a differential lncRNA-miRNA-mRNA regulatory network related to cell proliferation, differentiation and apoptosis and involved in stromal follicle development were established and preliminarily validated, which could be regarded as a key regulatory pathway of ovarian development in Yili geese.

**Supplementary Information:**

The online version contains supplementary material available at 10.1186/s12864-022-08774-4.

## Background

The development of ovary and follicle is an important factor affecting the laying performance of goose [1]. The ovarian volume of goose is smaller at non-reproductive period than that of the ovary during the laying period. No hierarchical follicles and many pre-hierarchical follicles could be detected of the ovaries at non-reproductive period. The ovary stay degenerated until the next egg-laying period [2]. The biological process of ovarian development and ovulation is transcriptionally regulated by a large number of key genes under dynamic and stage-specific expression [3]. Therefore, studying the expression characteristics of ovarian tissue at the transcriptional level in goose egg laying stages could provide an important basis for screening and identifying key genes regulating goose ovarian development. The Yili geese is a high-quality local characteristic species poultry in Xinjiang, China, are mainly characterized by strong adaptability, heat resistance, cold resistance, rough feeding resistance, and a certain flying ability. The Yili geese is one of the typical seasonal breeding animals and only has one egg laying cycle per year. This feature makes the Yili geese an ideal animal model to study the gene expression patterns in geese during each laying period.

Transcriptome of Zhedong white geese ovary at laying and nesting period showed that differential genes were mainly involved in norepinephrine metabolism, steroid hormone biosynthesis, Wnt signaling pathway, calcium signaling pathway, GnRH signaling pathway and oocyte meiosis, which were closely related to follicular development [4]. Based on high-throughput sequencing technology, the transcriptome sequencing of the gonadal axis tissues in high and low egg production Xinjiang Yili geese resulted in 30 candidate genes related to egg production [5]. However, the regulation mechanism, especially the non-coding RNA involved regulatory network of reproductive traits in goose is still unclear.

In this study, Yili geese at pre-laying, laying and ceased-laying period were selected for exploring the candidate genes and network that regulate the ovarian development by using RNA-seq and small RNA-seq technology. The results could provide new insights into the molecular regulatory mechanism of non-coding RNA-mediated ovary development in goose.

## Results

### Ovary histomorphometric analysis at different egg-laying periods in Yili geese

**Fig. 1:**
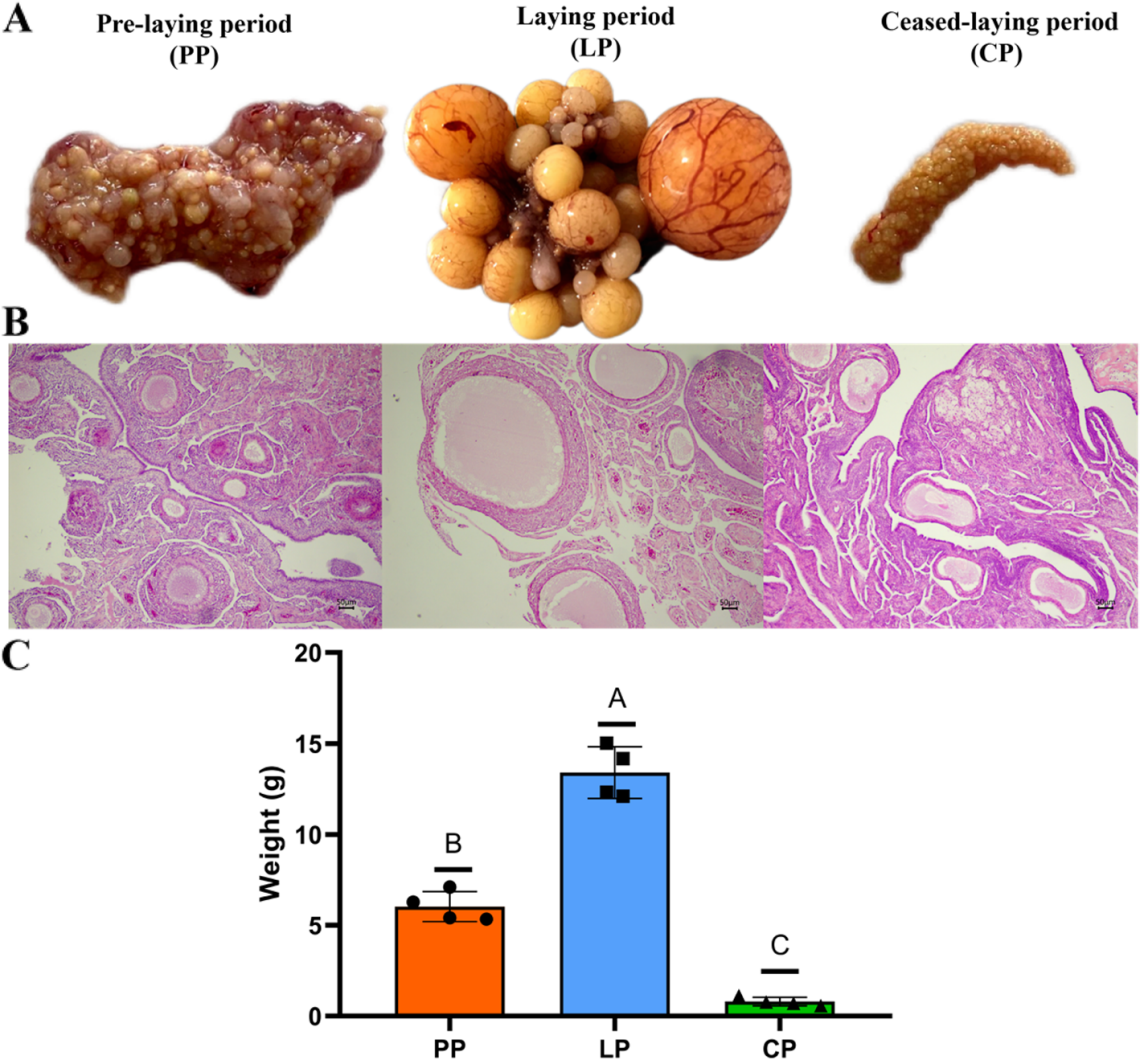
Histomorphometric of Yili geese ovary in different egg-laying periods. **A** Morphometric analysis of Yili geese ovary in different egg-laying periods. **B** Histological analysis of Yili geese ovary in different egg-laying periods. **C** Ovarian weight at each period

During the pre-laying period (PP), the ovaries of Yili geese showed primary follicles are numerous, while the ovaries during the laying period (LP) showed a hierarchy of follicles, and goose at ceased-laying period (CP) showed degenerated ovaries (Fig. 1A-B). Moreover, the ovarian weight showed highly significant differences between periods (*P* < 0.01) (Fig. 1C).

### Overview of sequencing and identification of lncRNA and miRNA

A total of 57,811,186 ~ 85,328,377 clean reads (Supplementary Table S1–1) were produced from the 12 ovary cDNA libraries, of which 80.85% ~ 87.21% sequences were uniquely mapped (Supplementary Table S1–2) to produce 38,296 lncRNA and 33,332 mRNA transcripts (Supplementary Table S1–3).

For miRNA libraries, a total of 69.38% ~ 93.34% clean reads were produced from the 12 ovary cDNA libraries (Supplementary Table S1–4), to produce 2700 known-miRNAs (Supplementary Table S1–5) and 2983 novel-miRNAs (Supplementary Table S1–6), and the proportion of annotated miRNAs was 47.51–74.58% (Supplementary Table S1–7). Most miRNA fragments are 18–25 nt in length, and the number of miRNAs with a length of 22 nt is the highest in each sample, with an average of 35.44% (Supplementary Table S1–8).

### Analysis of the differentially expressed mRNA, lncRNA and miRNA among different egg-laying periods

**Fig. 2:**
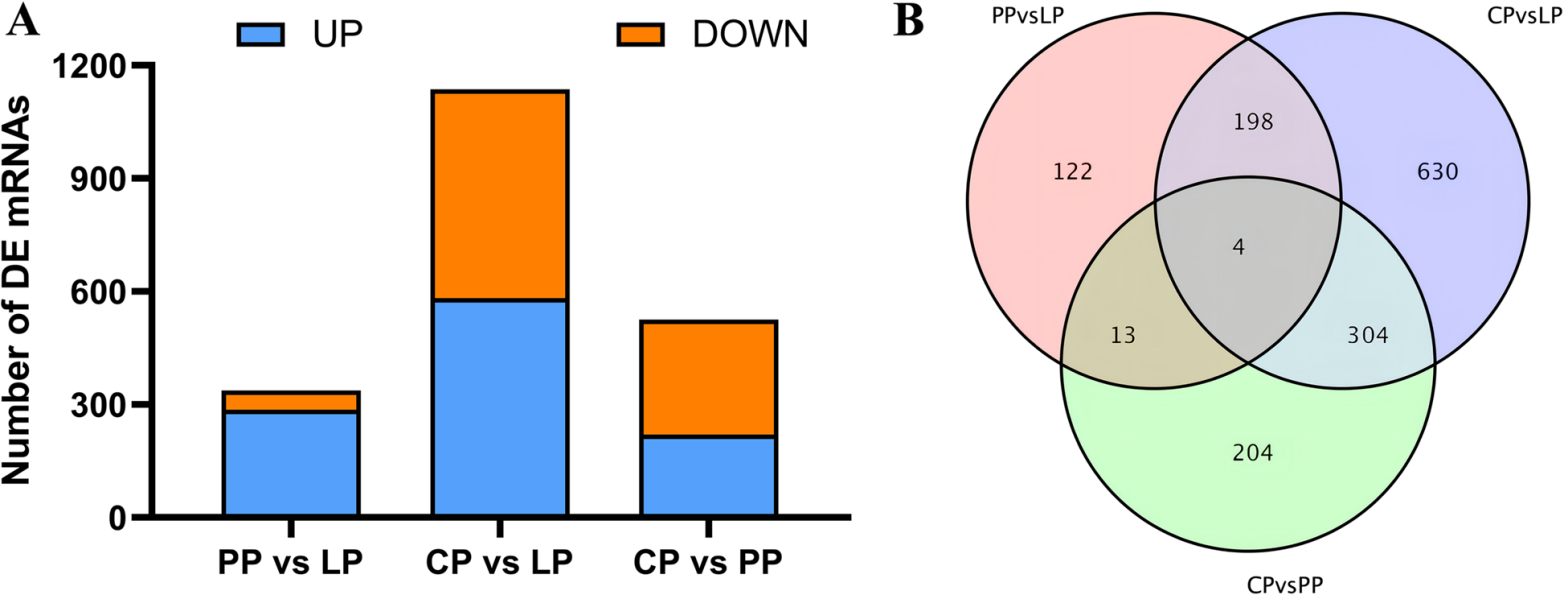
Differentially expressed mRNAs in ovary tissues of Yili geese in different egg-laying periods. **A** The number of DE mRNAs in different comparison group. **B** Venn diagrams of differentially expressed mRNA in different comparison group

A total of 337 mRNAs were differentially expressed (286 up-regulated and 51 down-regulated) in the LP as compared with PP (PP vs LP) (Fig. 2A and supplementary Table S2–1), 1136 mRNAs were differentially expressed (582 up-regulated and 554 down-regulated) in the LP as compared with CP (CP vs LP) (Fig. 2A and supplementary Table S2–2), and 525 mRNAs were differentially expressed (220 up-regulated and 305 down-regulated) in the PP as compared with CP (CP vs PP) (Fig. 2A and supplementary Table S2–3). Four differential genes were coexpressed in the three comparison groups (Fig. 2B and supplementary Table S2–4).

**Fig. 3:**
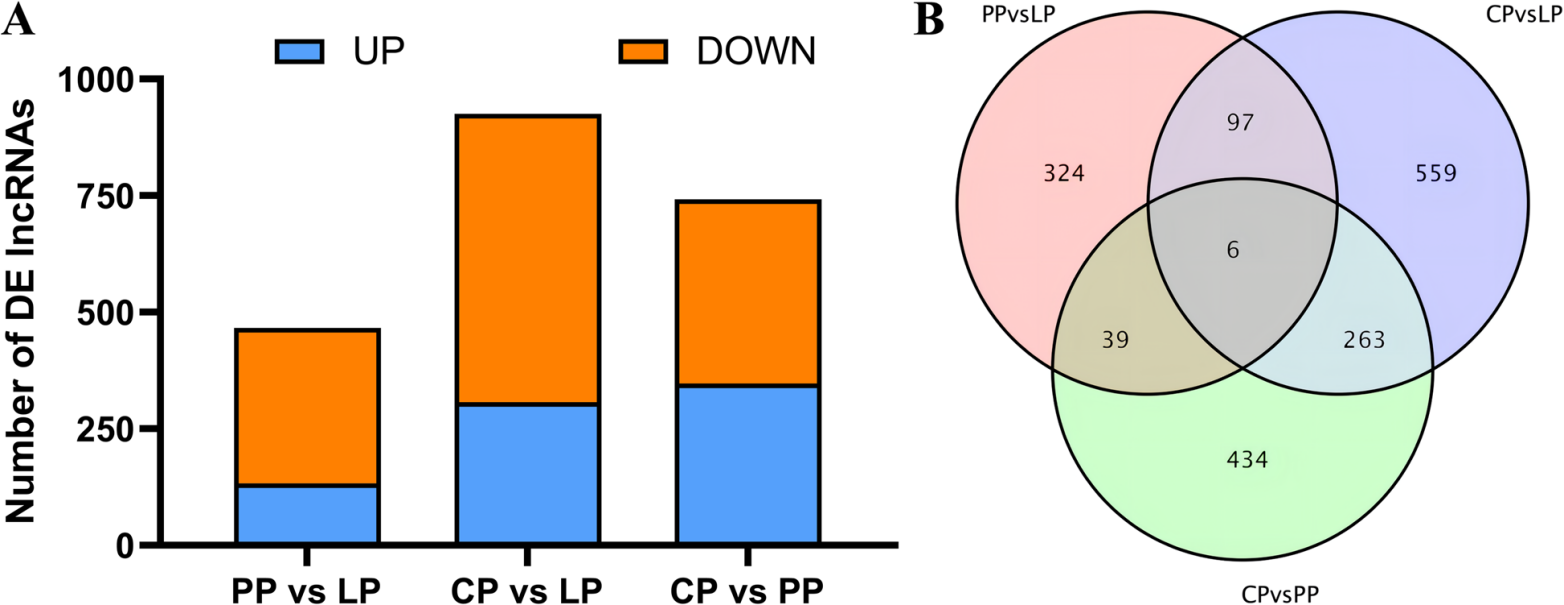
Differentially expressed lncRNAs in ovary tissues of Yili geese in different egg-laying periods. **A** The number of DE lncRNAs in different comparison group. **B** Venn diagrams of differentially expressed lncRNA in different comparison group

A total of 466 lncRNAs were differentially expressed (133 up-regulated and 333 down-regulated) in the LP as compared with PP (PP vs LP) (Fig. 3A and supplementary Table S2–5), 925 lncRNAs were differentially expressed (307 up-regulated and 618 down-regulated) in the LP as compared with CP (CP vs LP) (Fig. 3A and supplementary Table S2–6), and 742 lncRNAs were differentially expressed (347 up-regulated and 395 down-regulated) in the PP as compared with CP (CP vs PP) (Fig. 3A and supplementary Table S2–7). Six differential lncRNAs were coexpressed in the three comparison groups (Fig. 3B and supplementary Table S2–8).

**Fig. 4:**
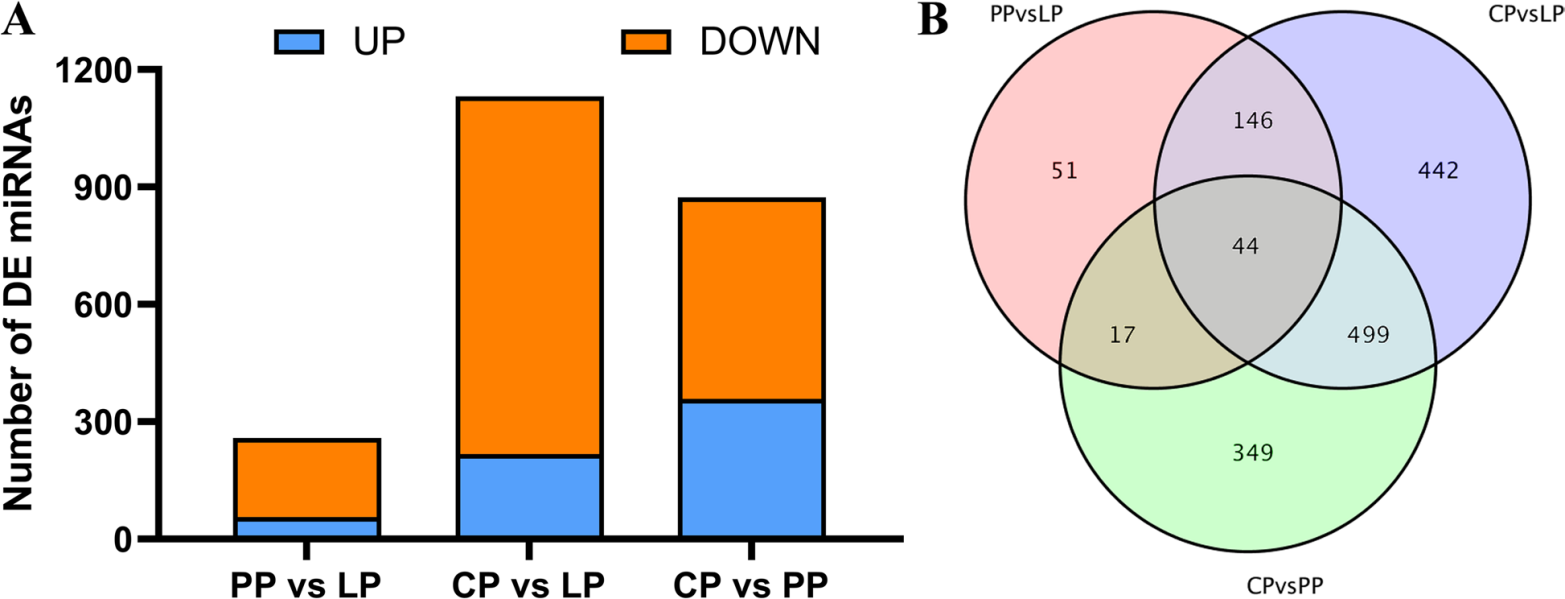
Differentially expressed miRNAs in ovary tissues of Yili geese in different egg-laying periods. **A** The number of DE miRNAs in different comparison group. **B** Venn diagrams of differentially expressed miRNA in different comparison group

A total of 258 miRNAs were differentially expressed (56 up-regulated and 202 down-regulated) in the LP as compared with PP (PP vs LP) (Fig. 4A and supplementary Table S2–9), 1131 miRNAs were differentially expressed (217 up-regulated and 914 down-regulated) in the LP as compared with CP (CP vs LP) (Fig. 4A and supplementary Table S2–10), and 909 miRNAs were differentially expressed (358 up-regulated and 515 down-regulated) in the PP as compared with CP (CP vs PP) (Fig. 4A and supplementary Table S2–11). Forty-four differential miRNAs were coexpressed in the three comparison groups (Fig. 4B and supplementary Table S2–12).

### Functional enrichment analysis of differentially expressed genes and target genes

**Fig. 5:**
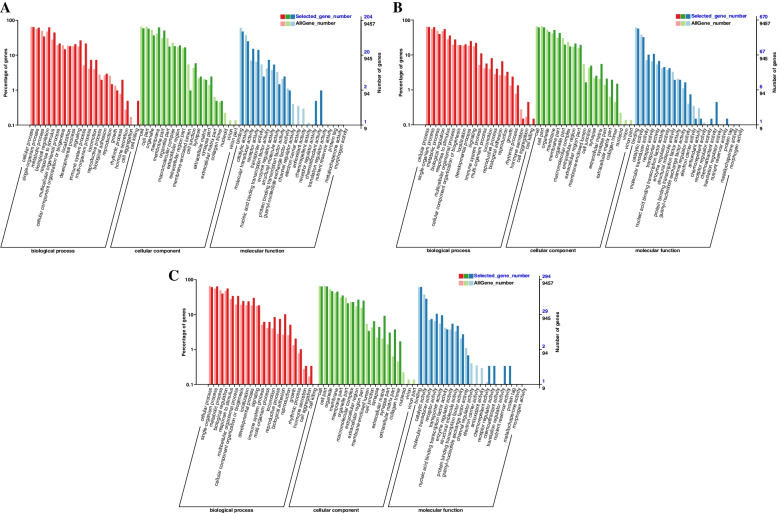
GO enrichment analysis of DE-mRNAs. **A** PP vs LP. **B** CP vs LP. **C** CP vs PP

In ovarian tissues of different egg-laying stages, most DEGs were enriched in the “biological process” and “cellular component” category. In the PP vs LP, CP vs LP, and CP vs PP groups, 204, 670, and 294 differential mRNAs were annotated by the GO database, respectively. In the PP vs LP, most of DEGs were enriched in cellular process (BP), biological regulation (BP), membrane (CC), cell (CC), and binding (MF) (Fig. 5A and supplementary Table S3–1). In the CP vs LP, most of DEGs were enriched in cellular process (BP), single-organism process (BP), cell (CC), cell part (CC), binding (MF) and catalytic activity (MF) (Fig. 5B and supplementary Table S3–2). In the CP vs PP, most of DEGs were enriched in single-organism process (BP), cellular process (BP), cell (CC), cell part (CC), binding (MF) and catalytic activity (MF) (Fig. 5C and supplementary Table S3–3). This indicates that most DEGs were related to cellular components and biological regulation process.

**Fig. 6:**
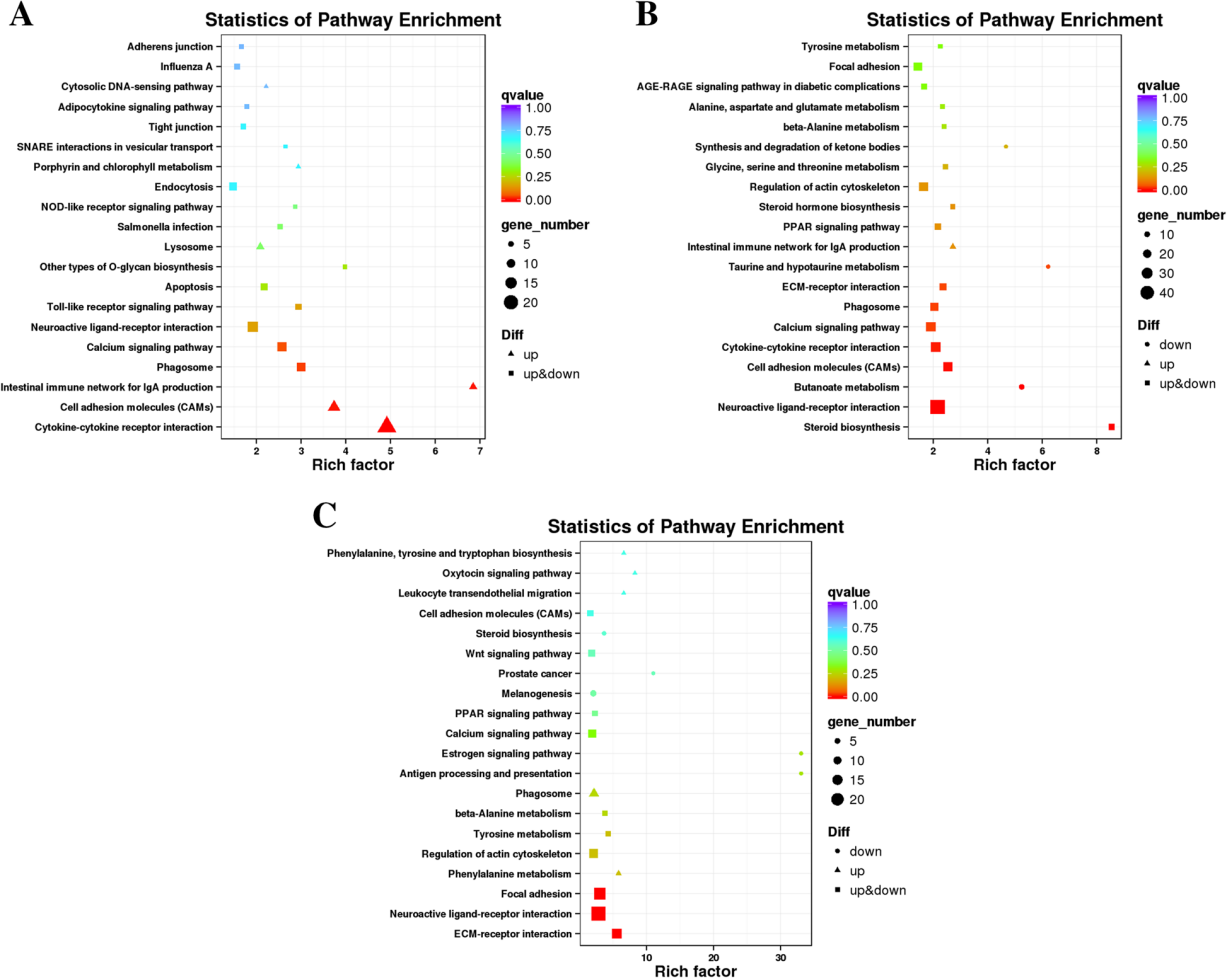
KEGG enrichment analysis of DE-mRNAs. **A** PP vs LP. **B** CP vs LP. **C** CP vs PP

Differential mRNAs were significantly enriched in 5, 9 and 3 pathways in PP vs LP, CP vs LP and CP vs PP groups, respectively (q-value< 0.05). In the PP vs LP group, the differentially enriched pathways including phagosome, calcium signaling pathway (Fig. 6A and supplementary Table S3–4). In the CP vs LP group, differentially enriched pathways including steroid biosynthesis, neuroactive ligand-receptor interaction (Fig. 6B and supplementary Table S3–5). In the CP vs PP group, differentially enriched pathways including ECM-receptor interaction, neuroactive ligand-receptor interaction, etc. (Fig. 6C and supplementary Table S3–6). Notably, the cytokine-cytokine receptor interaction, ECM-receptor interaction and calcium signaling pathway was commonly enriched by the DEGs in the ovarian tissues of different egg-laying stages.

**Fig. 7:**
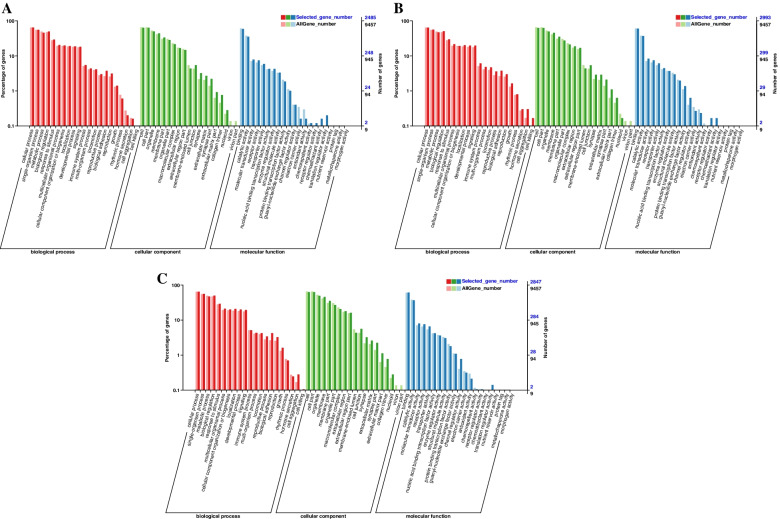
GO enrichment analysis of DE-lncRNAs target gene. **A** PP vs LP. **B** CP vs LP. **C** CP vs PP

It was found that 38,034 lncRNAs regulate 32,352 mRNAs in their adjacent positions by cis mode; 158 lncRNAs have potential targeting regulatory relationship with 12,939 mRNAs by trans mode. Based on the prediction results of lncRNAs target genes, this study conducted GO enrichment analysis on differentially expressed lncRNA target genes. It was found that 2485, 2993 and 2847 differential lncRNAs target genes in PP vs LP, CP vs LP and CP vs PP groups were annotated by GO database, respectively. In the PP vs LP, most of differential lncRNAs target genes were enriched in cellular process (BP), single-organism process (BP), cell (CC), organelle (CC), and binding (MF) (Fig. 7A and supplementary Table S4–1). In the CP vs LP, most of differential lncRNAs target genes were enriched in cellular process (BP), metabolic process (BP), cell (CC), membrane (CC), binding (MF) and catalytic activity (MF) (Fig. 7B and supplementary Table S4–2). In the CP vs PP, most of differential lncRNAs target genes were enriched in cellular process (BP), single-organism process (BP), cell (CC),organelle (CC), binding (MF) and catalytic activity (MF) (Fig. 7C and supplementary Table S4–3). This indicates that most of differential lncRNAs target genes were related to cellular component organization and biological regulation process.

**Fig. 8:**
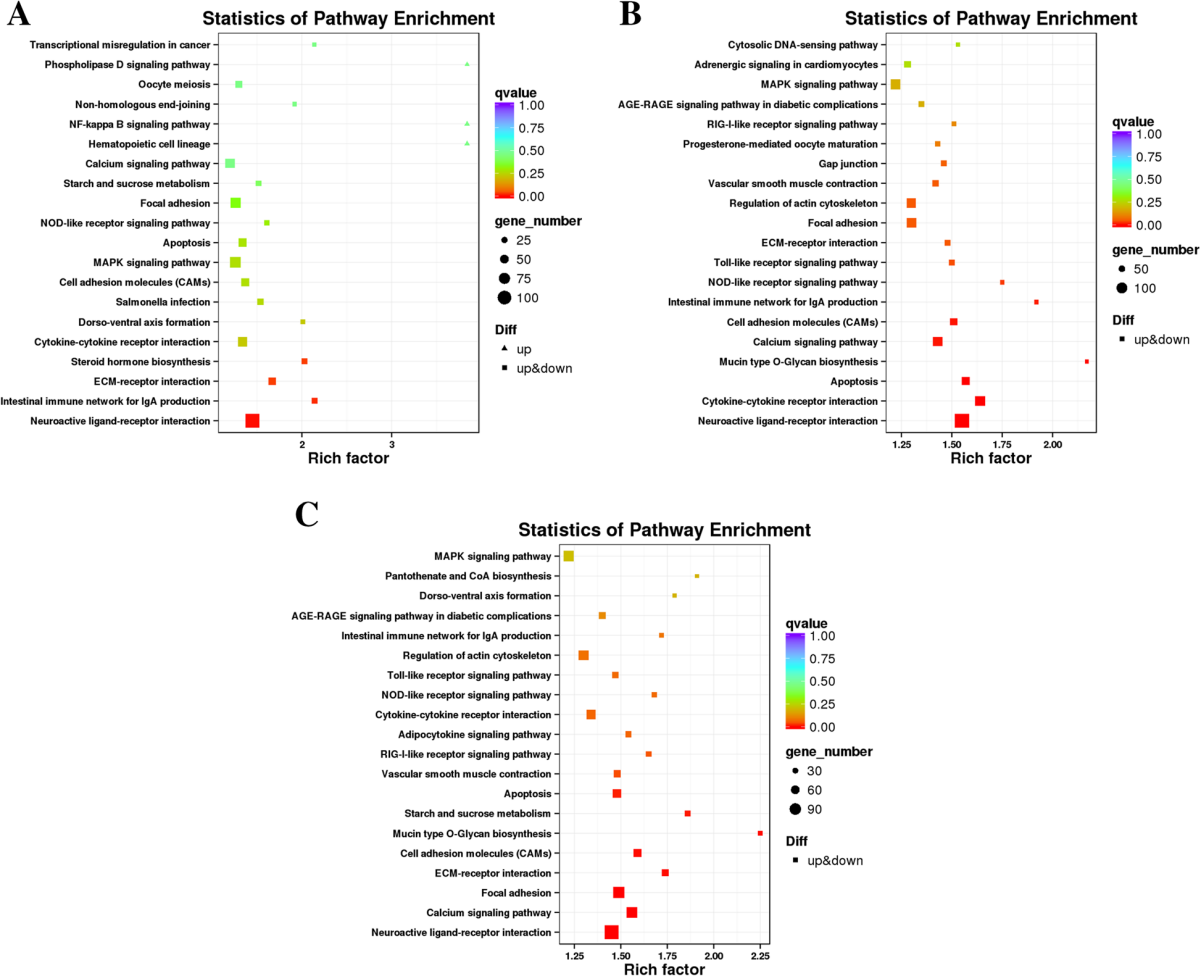
KEGG enrichment analysis of DE-lncRNAs target gene. **A** PP vs LP. **B** CP vs LP. **C** CP vs PP

Differential lncRNAs target genes were significantly enriched in 4, 12 and 10 pathways in PP vs LP, CP vs LP and CP vs PP groups, respectively (q-value< 0.05). In the PP vs LP group, the differentially enriched pathways including neuroactive ligand-receptor interaction, ECM-receptor interaction, etc. (Fig. 8A and supplementary Table S4–4). In the CP vs LP group, differentially enriched pathways including neuroactive ligand-receptor interaction, calcium signaling pathway, etc. (Fig. 8B and supplementary Table S4–5). In the CP vs PP group, differentially enriched pathways including neuroactive ligand-receptor interaction, calcium signaling pathway (Fig. 8C and supplementary Table S4–6). Notably, the neuroactive ligand-receptor interaction, ECM-receptor interaction apoptosis, and calcium signaling pathway was commonly enriched by the differential lncRNAs target genes in the ovarian tissues of different egg-laying stages.

**Fig. 9:**
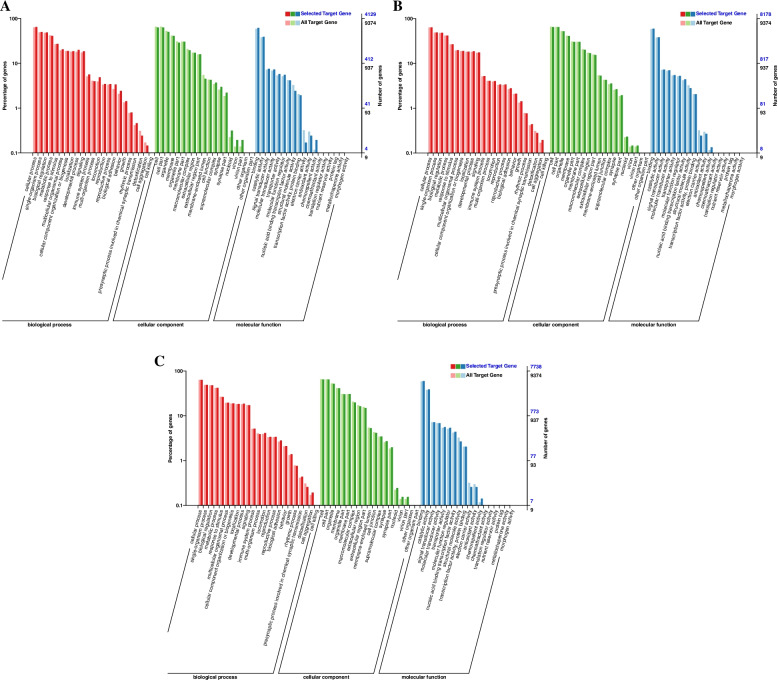
GO enrichment analysis of DE-miRNAs target gene. **A** PP vs LP. **B** CP vs LP. **C** CP vs PP

In the PP vs LP, CP vs LP and CP vs PP groups, 4129, 8178 and 7738 differential miRNAs target genes were annotated by the GO database, respectively. In the PP vs LP, most of differential miRNAs target genes were enriched in cellular process (BP), single-organism process (BP), organelle (CC), membrane (CC), and binding (MF) (Fig. 9A and supplementary Table S5–1). In the CP vs LP, most of differential miRNAs target genes were enriched in metabolic process (BP), single-organism process (BP), cell (CC), cell part (CC), binding (MF) and catalytic activity (MF) (Fig. 9B and supplementary Table S5–2). In the CP vs PP, most of differential miRNAs target genes were enriched in cellular process (BP), metabolic process (BP), cell (CC), organelle (CC) and binding (MF) (Fig. 9C and supplementary Table S5–3). This indicates that most of differential miRNAs target genes were related to cellular component organization and biological regulation process.

**Fig. 10:**
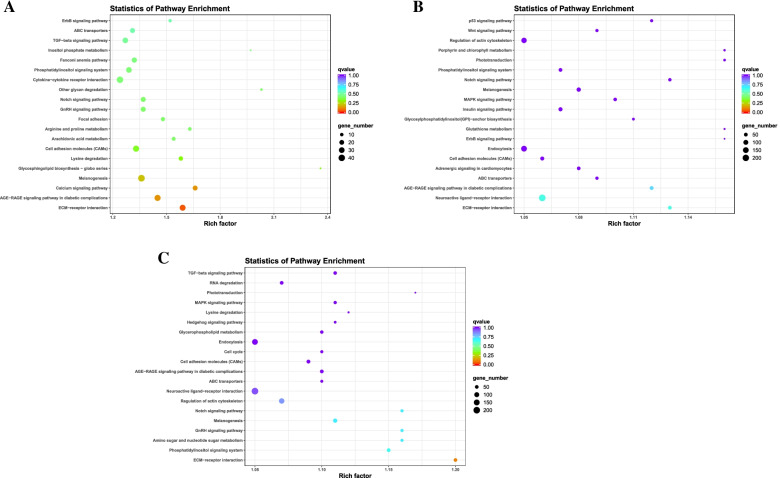
KEGG enrichment analysis of DE-miRNAs target gene**. A** PP vs LP. **B** CP vs LP. **C** CP vs PP

In the PP vs LP group, the differential miRNA target genes were significantly enriched in the ECM-receptor interaction (Fig. 10A and supplementary Table S5–4). No KEGG pathway was significantly enriched in the CP vs LP and CP vs PP groups. However, miRNAs target genes were involved in calcium signaling pathway, Notch signaling pathway and GnRH signaling pathway (Fig. 10B, C and supplementary Table S5–5 and 6). These results suggest that the ECM-receptor interaction pathway may play an important role in miRNA-mediated reproductive processes initiated during egg-laying.

### Construction of lncRNA-miRNA-mRNA networks and functional analysis

**Fig. 11:**
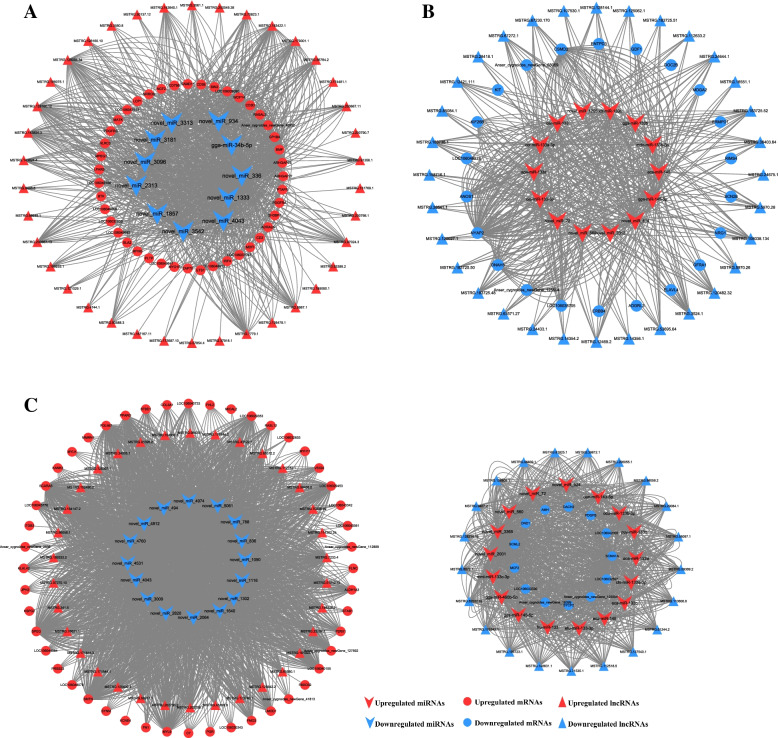
lncRNA–miRNA–mRNAs interaction network constructed and visualized. **A** PP vs LP. **B** CP vs LP. **C** CP vs PP. V shaped, circle, triangle denote miRNA, mRNA, and lncRNA, respectively. Red color represents up-regulation and blue represents down-regulation.

Elements from the differentially expressed RNAs were adopted to construct the ceRNA network of lncRNA-miRNA-mRNA. Each miRNA is negatively associated with multiple lncRNAs or mRNAs. The network of PP vs LP composed of 92 nodes and 1018 edges, and the nodes included 38 lncRNAs, 11 miRNAs and 43 mRNAs (Fig. 11A and supplementary Table S6–1). In the network of PP vs LP group, miRNA expression was significantly down-regulated, while mRNA and lncRNA expression were both significantly up-regulated, and central nodes include novel_miR_336, novel_miR_1333, gga-miR-34b-5p, *WDFY4*, *PDGFRB* and MSTRG.129094.34, etc. The network of CP vs LP composed of 68 nodes and 496 edges, and the nodes included 32 lncRNAs, 14 miRNAs and 22 mRNAs (Fig. 11B and supplementary Table S6–2). In the network of CP vs LP group, miRNA expression was significantly up-regulated, while mRNA and lncRNA expression were both significantly down-regulated, and central nodes include gga-miR-145–5p, eca-miR-145, *ANOS1*, *ERBB4*, MSTRG.3524.1, and MSTRG.5970.28, etc. The network of CP vs PP composed of 149 nodes and 3882 edges, and the nodes included 55 lncRNAs (34 up-regulated lncRNAs and 21 down-regulated lncRNAs), 33 miRNAs (17 up-regulated miRNAs and 16 down-regulated miRNAs) and 61 mRNAs (48 up-regulated mRNAs and 13 down-regulated mRNAs) (Fig. 11C and supplementary Table S6–3). The network central nodes of the CP vs PP group include novel_miR_2064, novel_miR_1116, gga-miR-145–5p, *MYL9* and MSTRG.164147.2, etc. Notably, gga-miR-145–5p, eca-miR-145, novel_miR_560, and novel_miR_72 in the ceRNA network were participated in CP vs LP and CP vs PP groups. Meanwhile, the above miRNAs were significantly up-regulated in both the CP vs LP and CP vs PP groups.

**Fig. 12:**
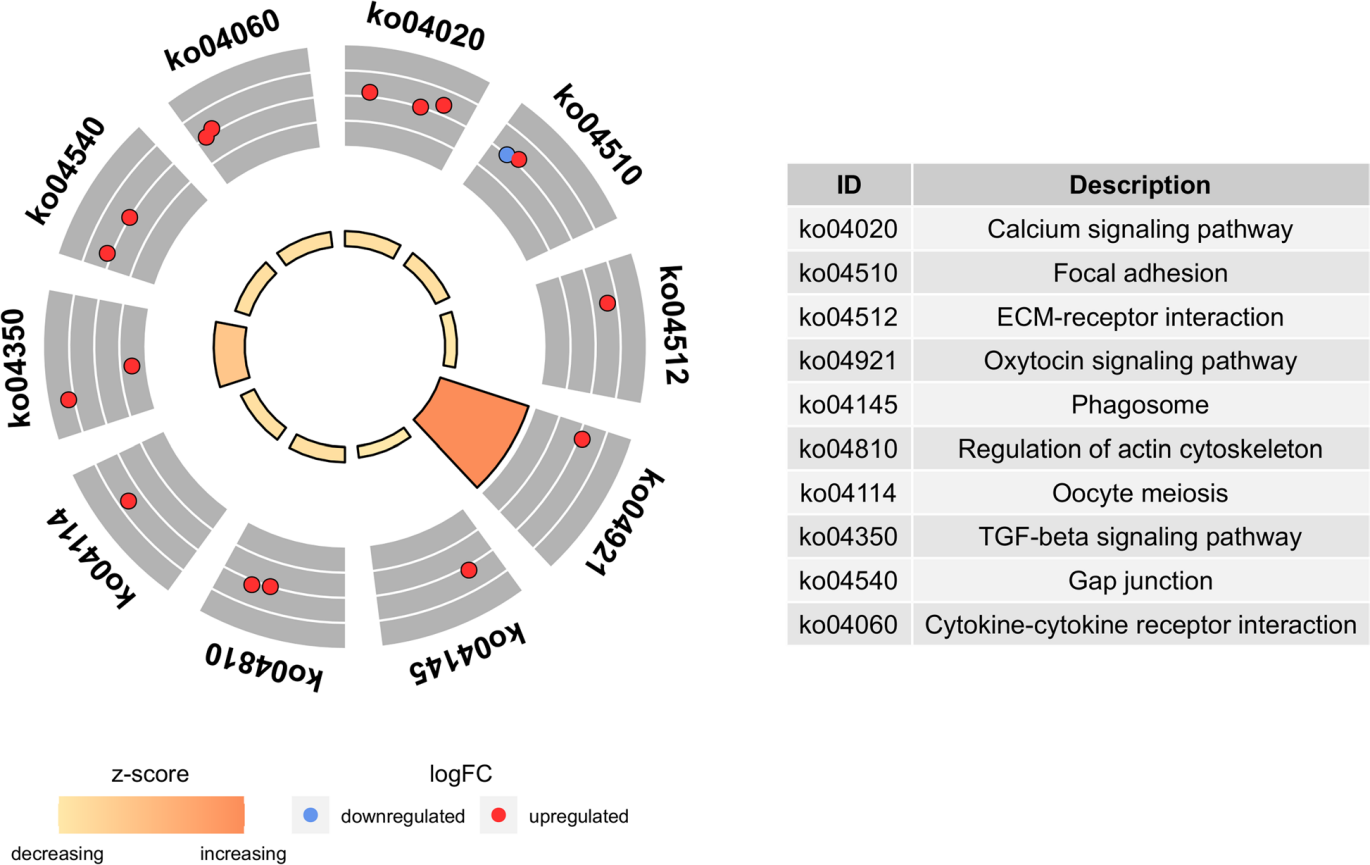
KEGG enrichment analysis of mRNAs in the ceRNA regulatory network

Furthermore, functional enrichment analyses of mRNA involved in ceRNA network were deeply analyzed (Fig. 12). Genes were mostly enriched in the calcium signaling pathway, ECM-receptor interaction, oxytocin signaling pathway and oocyte meiosis pathways.

### Validation of RNA-Seq results

**Fig. 13:**
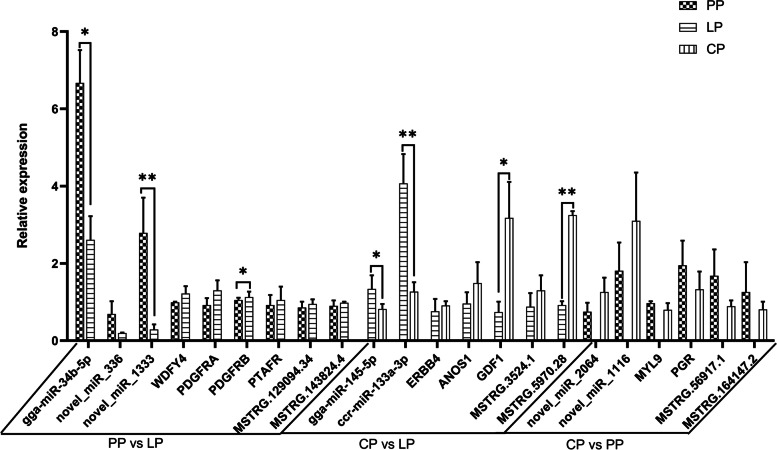
RT-qPCR verification of key nodes in ceRNA network

In the lncRNA-miRNA-mRNAs interaction network, the expression levels of 9, 7, and 6 key nodes in the PP vs LP, CP vs LP, and CP vs PP groups, respectively, were verified by RT-qPCR. (Fig. 13). The results showed that the expression trends of key node genes in each group were consistent with the RNA-seq data, indicating that the RNA-seq data were reliable. And the expression trend of miRNA in each group was opposite to that of its target lncRNA and mRNA. The above results showed that the gene expression relationship of these 22 key nodes was in line with the ceRNA hypothesis. These key node genes may be one of the important reasons affecting the ovarian development of Yili geese.

### Verification of the interaction among MSTRG.5970.28, gga-miR-145–5p, and *ERBB4*

**Fig. 14:**
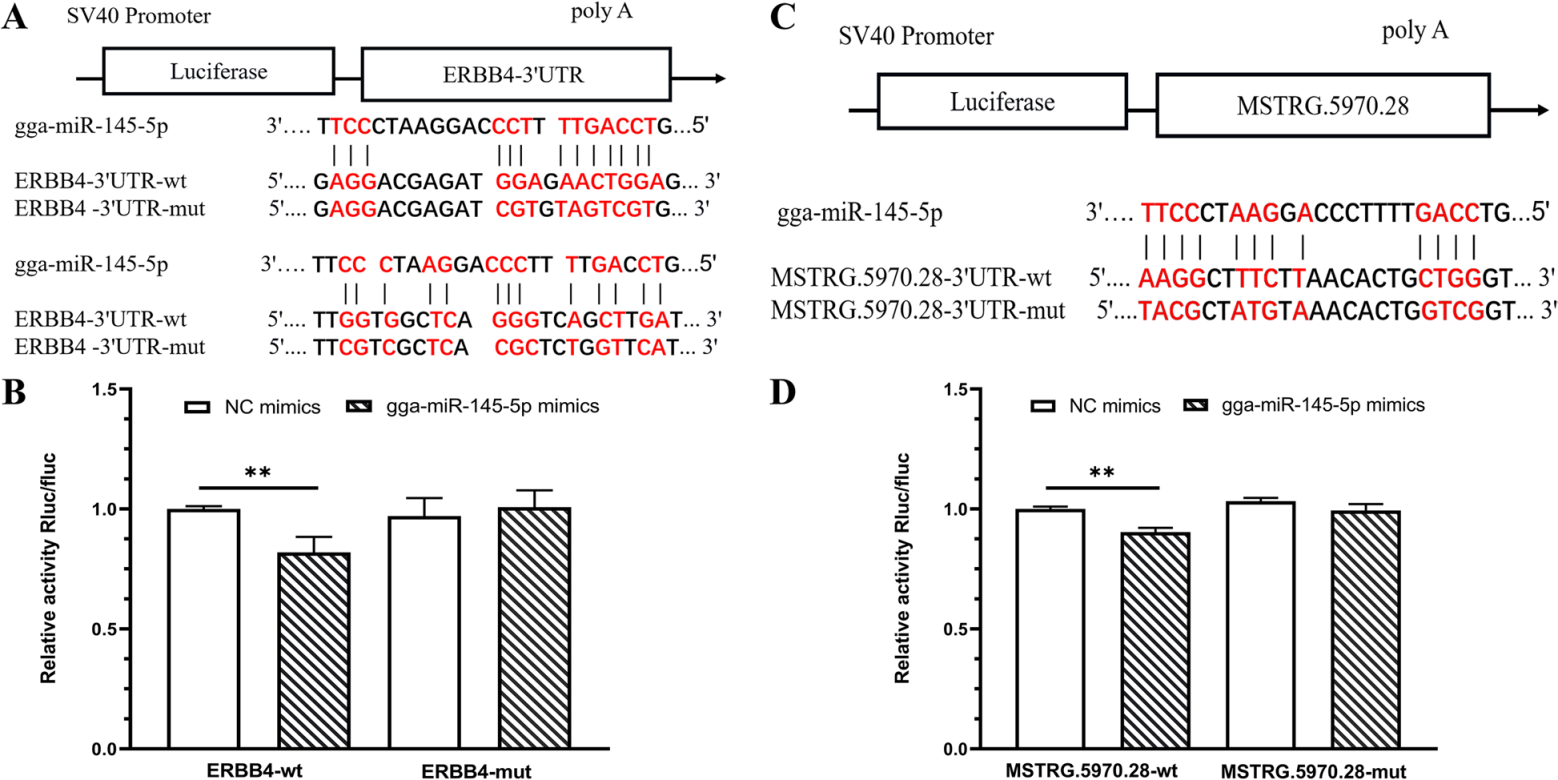
MSTRG.5970.28 bind to gga-miR-145–5p. **A** The predicted binding site and mutated site of gga-miR-145–5p in *ERBB4*. **B** Detection of interaction between *ERBB4* and gga-miR-145–5p by dual luciferase reporter gene assay. **C** The predicted binding site and mutated site of gga-miR-145–5p in MSTRG.5970.28. **D** Detection of interaction between MSTRG.5970.28 and gga-miR-145–5p by dual luciferase reporter gene assay

MSTRG.5970.28 and *ERBB4* were downregulated in the CP vs LP group, while gga-miR-145–5p was upregulated in the CP vs LP group. Targetscan and miRanda software predicted that MSTRG.5970.28 and *ERBB4* was targeted to gga-miR-145–5p (Fig. 14A, C). Therefore, mutant vectors were constructed to verify the specific binding sites. The results showed that compared with the NC group, gga-miR-145–5p significantly decreased the expression of luciferase in MSTRG.5970.28-wt (*P* < 0.01) and ERBB4-wt (*P* < 0.01), and no significant effect on luciferase activity when co-transfected with mutated plasmids (Fig. 14B, D). The results suggest that MSTRG.5970.28 and *ERBB4* directly targets gga-miR-145–5p.

## Discussion

The ovary is an important reproductive organ of female animals, its main function is to produce and discharge healthy eggs that could be fertilized, and to secret sex hormones to maintain the sexual characteristics and periodic reproductive activities [6]. The development of animal ovary, especially follicle development and ovulation biology, is distinctly stage-specific and regulated by their own gene expression [3]. Studies have shown that mRNA and non-coding RNA have important regulatory roles in reproductive-related processes such as gonadal development, hormone regulation, sex determination, and embryo implantation [7–11].

In the present study, significant differences in both the morphology, histology and weight of ovarian tissues were observed of Yili geese at different egg-laying periods. Consistent with this observation, 337, 1136, and 525 DEGs were identified in the PP vs LP, CP vs LP, and CP vs PP groups, respectively. Taken together, these identified DEGS may be can potentially be used to explain the specific functions that regulate ovarian development in geese. There were four differentially expressed genes appeared in all the three comparison groups, namely *ADRA2A*, *CP*, *GPNMB* and *LOC106033756*. Notably, these four overlapping differential mRNAs and the remaining 178 differential mRNAs were significantly up-regulated when geese enters the laying period from pre-laying period, and significantly down-regulated when geese enters the ceased-laying period from the laying period (Supplementary Table S7–1). The high expression of the above differential mRNAs may promote the ovarian development process. In addition, 180 differential mRNAs were significantly up-regulated when the goose from the laying to the ceased-laying period and significantly down-regulated when the goose from the ceased-laying to the pre-laying period (Supplementary Table S7–2), suggesting that the high expression of these differential mRNAs may have inhibite the ovarian development process. Some of them have been reported to be associated with ovarian development or reproductive processes, such as *GPNMB*, *CXCR4*, *HGF* and *PDGFRB*. On a physiological level, *GPNMB* improves cell invasion and motility, enabling metastasis. *GPNMB* was found to be highly expressed in breast cancer samples [12]. Elevated *CXCR4* have been reported to promote the occurrence of endometriosis [13], suggested its relation to the regulation of ovary function. *HGF* plays a promoting role in estrogen-mediated stimulation of ovarian cell growth and differentiation [14]. Elevated *PDGFRB* may promote steroid hormone synthesis in mouse ovaries [15].

To further reveal the biological implications of these identified DEGs, GO annotation and KEGG enrichment analysis were performed. Most of the DEGs in ovarian tissues of different egg-laying stages were enriched in the GO terms related to cell part, membrane, binding and catalytic activity process, suggesting that cellular components and biological regulation process could be essential for the ovarian development of geese. The results of KEGG enrichment analysis showed that cytokine-cytokine receptor interaction, cell adhesion molecules (CAMs), ECM-receptor interaction, phagosome, neuroactive ligand-receptor interaction, and calcium signaling pathway were enriched in two or three comparison groups. Differential genes in significantly enriched pathways may be associated with ovarian development, such as *ERBB4* and *LHCGR*. *ERBB4* was significantly downregulated in the CP vs LP group. Loss of *ERBB4* gene may cause aberrant ovarian function by affecting reproductive function and metabolic function [16]. *LHCGR* was significantly downregulated in both CP vs LP and CP vs PP group. The expression of *LHCGR* is associated with ovulation and luteinization of the ovary [17]. It is suggested that the differentially expressed genes screened in this study may play a key regulatory role in the ovarian development of Yili geese by affecting ovarian function.

The regulation of follicle and oocyte maturation is a complex multi-factor regulation process that requires specific genes to be accurately expressed at a specific time [3]. Studies have shown that lncRNA has an important regulatory role in reproduction-related processes such as gonadal development, hormone regulation, sexual determination, meiosis, and embryo implantation [18, 19]. By analyzing the lncRNAs expression profiles of ovarian tissues of Yili geese at different egg-laying periods, we identified 51 differential lncRNAs that were significantly up-regulated when the geese entered the laying period from the pre-laying and significantly down-regulated when the geese entered the ceased-laying from the laying period (Supplementary Table S7–3), suggesting that the high expression of these differential lncRNAs may promote the developmental process of the ovaries. In addition, 79 differential mRNAs were significantly up-regulated when the goose from the laying to the ceased-laying period and significantly down-regulated when the goose from the ceased-laying to the pre-laying period (Supplementary Table S7–4), suggesting that the high expression of these differential lncRNAs may have inhibite the ovarian development process. Six lncRNAs were differentially expressed in all three comparison groups, including MSTRG.111093.1, MSTRG.119742.1 and MSTRG.127167.95, et al. And the target genes of the above six differential lncRNAs were significantly enriched in reproduction-related calcium signaling pathways [20], apoptosis [21] and ECM-receptor interactions [22], suggested that the six differential lncRNAs could affected the ovarian development of Yili geese by regulating the target genes in the above-mentioned signaling pathways.

The GO term with significant enrichment of differential lncRNAs target genes was mainly related to biological regulation, cellular process, metabolic process. The KEGG enrichment analysis showed that apoptosis, calcium signaling pathway, cell adhesion molecules (CAMs), and ECM-receptor interaction pathways were significantly enriched in two or three comparison groups. During reproduction, preventing cell apoptosis and autophagy could obtain high-quality oocytes in the ovary, maintain ovarian function, and protect female fertility [21]. High expression of *CTSS* in ovary induced excess lipid deposit, oxidative stress and potential ovulation damage [23]. High level of *FAS* in antral follicle initiated ovarian cell death [24]. *RIPK1*, *FADD*, *TRADD* and *TNFRSF1A* were all highly expressed in cell apoptosis [25], which would also promote cell apoptosis in the ovary. In this study, 23, 8, 4, 2, 6, 16 differential lncRNAs targeted *CTSS*, *FAS*, *RIPK1*, *FADD*, *TRADD*, and *TNFRSF1A* (Supplementary Table S8–1), respectively, which were significantly enriched in the apoptosis pathway, indicating that the above differential lncRNAs might inhibit ovarian development of Yili geese. It is well known that intracellular Ca^2+^ concentration is a key signaling molecule to control exocytosis and regulate the release of neurotransmitters and endocrine hormones [20]. Wang et al. [26] found that *EGFR* and *GNAS* were involved in the reproductive process of pigs. In this study, 4 and 1 differential lncRNAs were found to be significantly enriched in the calcium signaling pathway by targeting *EGFR* and *GNAS*, respectively. It is speculated that the above 5 differential lncRNAs participate in the development of the ovary of Yili geese by regulating the release of neurotransmitters and endocrine hormones. In addition, 23, 8, 7 and 24 differential lncRNAs were found in this study to target *HGF*, *PDGFRB*, *ERBB4* and *LHCGR* (Supplementary Table S8–2), respectively, which were reported to regulate ovarian function [14–17].

Studies have found that miRNA has an important function in the development of the mouse ovary, including meiosis of oocytes, reproductive regulation [27]. By analyzing the miRNA expression profiles of ovarian tissues of Yili geese at different egg-laying periods, we found that the number of differential miRNAs was higher in CP vsLP than in PP vs LP and CP vs LP groups, and this expression trend was similar to that of differential mRNAs and differential lncRNAs. Interestingly, we identified 35 differential miRNAs that were significantly up-regulated when the geese enters the laying period from pre-laying period, and significantly down-regulated when geese enters the ceased-laying period from the laying period (Supplementary Table S7–5), suggesting that the high expression of these differential miRNAs may promote the developmental process of the ovary. In addition, 363 differential miRNAs were significantly up-regulated when the goose from the laying to the ceased-laying period and significantly down-regulated when the goose from the ceased-laying to the pre-laying period (Supplementary Table S7–6), suggesting that the high expression of these differential miRNAs may have inhibite the ovarian development process. There were 44 differential miRNAs overlapped in the three comparison groups, which were involved in ECM-receptor interaction [22], FoxO signaling pathway [28], and oxytocin signaling pathway [29] related to reproductive regulation.

KEGG enrichment analysis showed that miRNAs target genes in the PP vs LP group were significantly enriched in ECM-receptor interaction, in which *THBS1* was involved in mediating IGF-I-induced steroid production and proliferation in granulosa cells during follicular development [30]. *TNC* defective would cause the failure of ovulation [31]. In this study, novel_miR_1844, novel_miR_1333, and novel_miR_336 target *THBS1* and *TNC*, respectively, might regulate the ovulation process of Yili geese.

In this study, oha-miR-375–3p, tgu-miR-375, and gga-miR-375 were significantly different in the PPvs LP group. Compared with the pre-laying period, it was increased by 46.8 times during the laying period. Studies have shown that the Wnt signaling pathway was regulated by miR-375 [32], and the Wnt signaling pathway is crucial in the differentiation, proliferation and metastasis of primordial germ cells and the regulation of stromal cells [33]. This indicated that oha-miR-375–3p, tgu-miR-375, and gga-miR-375 might participate in the regulation of the developmental process of the ovaries from the prelaying period to the laying period through the Wnt signaling pathway. In the CPvs LP group, the top 10 foldchanged novel_miR_1116, novel_miR_1473, novel_miR_2688, novel_miR_3346, novel_miR_3441, novel_miR_3838, novel_miR_4002, novel_miR_4129 and novel_miR_4382 target genes participate mainly in hedgehog signaling pathway, MAPK signaling pathway, and FoxO signaling pathway. Hedgehog signaling pathway was involved in regulating the proliferation and differentiation of germline stem cells [34]. MAPKs mediate signal transmission related to a variety of cell activities in cells, including cell proliferation, differentiation, survival, death and transformation [35, 36]. The main function of FoxO is to regulate cell cycle, apoptosis, atrophy and energy balance [37]. Therefore, these novel miRNAs might play a key role in regulating the proliferation and differentiation of germline stem cells and signal transmission related to cell activities. In the CP vs PP group, gga-miR-34c-5p and ppa-miR-34c were significantly down-regulated with 71.4-fold and 71.2-fold, respectively. Members of the miR-34 family (miR-34a, miR-34b, and miR-34c) have been widely speculated to be key mediators of p53 pathway [38, 39], which regulated a variety of cellular processes, including apoptosis, senescence, cell cycle arrest, differentiation, and DNA repair and replication [40]. It is speculated that gga-miR-34c-5p and ppa-miR-34c might regulate the development, senescence and apoptosis of ovarian cells.

Through the miRNA binding elements, lncRNA could compete with mRNA to combine with miRNA, so as to realize the regulation of lncRNA on mRNA expression [41]. Despite great progress in understanding human disease using the ceRNA, relatively few studies have been carried out in the regulation of ovarian development and reproductive traits. By analyzing the differential mRNAs of different egg-laying stages, we obtained some important candidate genes associated with ovarian development. Interestingly, genes such as *PDGFRB, WDFY4, PDGFRA* and *PTAFR* were also identified in the ceRNA network of the PP vs LP group. Novel_miR_336 showed the most nodes in the regulatory network, and its expression was down-regulated in the PP vs LP group. The predicted target genes, *PDGFRB* and *LPAR4*, were negatively correlated with the expression of novel_miR_336. *PDGFRB* was involved in controlling the synthesis of steroid hormones in the ovaries of mice [15]. *LPAR4* is expressed at high levels in human ovaries [42]. In this network, there were many nodes of MSTRG.129094.34, and its target genes were involved in a number of pathways related to reproduction, such as MAPK signaling pathway and Calcium signaling pathway. At the same time, MSTRG.129094.34 was predicted to target novel_miR_336, and to be negatively regulated by novel_miR_336. It is speculated that MSTRG.129094.34 might up-regulate *PDGFRB* and *LPAR4* by targeting novel_miR_336, thereby participating in the synthesis of steroid hormones in the ovary. Studies have shown that mmu-miR-34b-5p and mmu-miR-107–3p might be involved in the alternative splicing of kitl pre-mRNA in mouse ovarian granulosa cells [43]. *WDFY4* was associated with antigen processing, T cell activation, and immune response in humans, mice, and rats [44]. MSTRG.129094.34 targeted gga-miR-34b-5p which further regulated the expression of *WDFY4* to promote the immune response in the ovary, and then affecting the ovarian development process.

In the ceRNA network of CP vs LP group, the expression of both differential mRNAs and lncRNAs were down-regulated, and the expression of differential miRNA were up-regulated. In the ceRNA network of the CP vs LP group, we noted the inclusion of screened candidate genes for ovarian development, such as *KIT*, *KIF26B* and *ELAVL4*. Novel_miR_814 has the most nodes in the regulatory network, and its expression was up-regulated in the CP vs LP group, which targeted to *NRG1* and *KIT* and showed negative correlation with this two genes. The expression of *NRG1* was related to LH-induced ovulation and depends on the ERK1/2-C/EBP regulatory pathway. It is supposed that *NRG1* could enhance granulosa cell luteinization and regulate the maturation and development of oocytes [45]. *KIT* was related to the cleavage of vitelline membrane and the formation of primordial follicles and the number of oocytes [46]. LncRNA of MSTRG.128144.1 and MSTRG.158716.1 targeted to *NRG1* and *KIT,* respectively, through trans-action. It is speculated that MSTRG.128144.1 and MSTRG.158716.1 could release the inhibitory factor on the target genes of *NRG1* and *KIT,* respectively, by adsorbing novel_miR_814. The other two miRNAs of gga-miR-145–5p and ccr-miR-133a-3p have multiple nodes in this network. Overexpression of miR-145–5p could inhibit the activation of Notch signaling pathway and apoptosis signaling pathway [47], which regulated cell proliferation, differentiation and apoptosis, and participated in the development of stromal follicles [48]. Both gga-miR-145–5p and MSTRG.5970.28/MSTRG.3524.1 targeted *ERBB4*. *ERBB4* has been demonstrated to be related to ovarian function. Thus, it is speculated that MSTRG.5970.28/MSTRG.3524.1 might be act as the ceRNA sponges of gga-miR-145–5p and affect the expression of *ERBB4*. MiR-133 has been demonstrated to regulate oocyte meiosis [49] and suppress ovarian cancer cell proliferation [50, 51]. *NYAP2* and MSTRG.24644.1 were significantly down-regulated in the CP vs LP group, and both targeted to ccr-miR-133a-3p. It is speculated that MSTRG.24644.1 affects the expression of *NYAP2* by acting as the ceRNA sponges of ccr-miR-133a-3p. Moreover, the results of dual-luciferase reporter detection suggested that there were target sites of gga-miR-145–5p in the MSTRG.5970.28 sequence and *ERBB4* mRNA 3′-UTR. It is suggested that the MSTRG.5970.28 may regulates *ERBB4* expression by binding gga-miR-145–5p, and then regulates the growth and development of Yili geese ovary.

In the ceRNA network of CP vs PP group, there are two modes of regulation between lncRNA, miRNA and mRNA. Novel_miR_2064 had the most interaction relationships in the regulatory network and was significantly downregulated in the CP vs PP groups. The novel_miR_2064 and MSTRG.7233.4 was predicted to target gene MYL9, which is enriched in the oxytocin signaling pathway. It is speculated that MSTRG.7233.4 affects the expression of *MYL9* by acting as a ceRNA of novel_miR_2064. Notably, the differential gene expression analysis of ovarian tissues of Yili geese at different egg-laying stages revealed that high expression of *SYCP2*, *LOC106042005* and *MCF2* genes might inhibit ovarian development. *SYCP2* is expressed in both testis and ovary and is essential for male fertility [52]. *MCF2* was found to be expressed in bovine oocytes/ovaries and testis [53]. In the ceRNA network of the CPvsPP group, the expression of *SYCP2*, *MCF2* and *LOC106042005* was significantly downregulated and targeted to novel_miR_2001 and 21 lncRNAs. In addition, oan-miR-143–5p and gga-miR-145–5p in this network have been reported to be related to the reproduction process, in which oan-miR-143–5p belongs to the miR-143 family, while miR-143 is related to steroid hormone synthesis, proliferation and apoptosis of granulosa cells [54, 55]. There were 21 lncRNAs targeted to PDGFB and oan-miR-143–5p, 22 lncRNAs targeted to *LOC106032597* and gga-miR-145–5p in this network. Therefore, the lncRNAs-miRNAs-mRNA regulatory network constructed in this study might affect steroid hormone synthesis, granulosa cell proliferation and apoptosis, and then affect the ovarian development of Yili geese. Despite our observations, the underlying mechanisms need further investigation.

## Conclusions

In this study, differentially expressed mRNAs, lncRNAs and miRNAs were identified in the ovary of Yili geese at different egg-laying stages. It was found that differential mRNAs, lncRNAs and miRNAs were mainly related to cellular processes, biological regulation, ECM-receptor interaction, neuroactive ligand-receptor interaction, and calcium signaling pathway. A differential lncRNA-miRNA-mRNA regulatory network related to cell proliferation, differentiation and apoptosis and involved in stromal follicle development were established and preliminarily validated, which could be regarded as a key regulatory pathway of ovarian development in Yili geese.

## Materials and methods

### Animals, sample collection and Histomorphometric analysis

Yili geese enters the peak-laying period at the third year. In this study, 4 geese at 3-year-olds at the same batch and similar body weight (3.2 kg ± 0.2 kg) were selected at the period of pre-laying (39 months) (PP), laying (41 months) (LP) and ceased-laying period (46 months) (CP), respectively. All the geese were provided by the National Yili geese breeding farm (Xinjiang, China). All selected geese were euthanized by inhaling carbon dioxide and cervical dislocation, which performed by competent personnel who experienced and correctly applied the technique. Then, the ovary removed immediately after slaughter. The morphological characteristics and weight data of the ovary were recorded quickly. Stroma with cortical follicles < 2 mm in diameter were dissected out of the ovaries, rinsed with PBS buffer and fixed in 4% paraformaldehyde. The samples were then embedded in paraffin, sectioned (5 μm) and mounted on slides, and standard hematoxylin and eosin (H&E) staining was performed. Histological characteristics of the ovarian stroma were observed using Nikon Eclipse Ci-L instrument and Nikon E.Z-MET software. For RNA-seq, collected ovaries were stored in liquid nitrogen and then transferred to − 80 °C.

### RNA extraction, RNA-seq library preparation, and sequencing

Total RNA was extracted from each ovarian tissue using TRIzol reagent (Invitrogen, Carlsbad, CA, USA). RNA degradation and contamination were detected by 1.5% agarose gel. The purity and concentration of RNA were determined from OD260/280 readings using the Nanodrop 2000 (Thermo Fisher Scientific Inc., Waltham, MA, USA). RNA integrity was checked using Agilent 2100 Bioanalyzer (Agilent Technologies,Palo Alto, CA, USA).

Approximately 3 μg RNA per sample was used for mRNA and lncRNA libraries. Ribosomal RNA was depleted using the Ribo-Zero™ rRNA Removal Kit (Epicentre, Madison, USA). After rRNA depletion, the remaining RNA was purified. The NEBNext® Ultra™ The Directional RNA Library Prep Kit for Illumina® (New England Biolabs; NEB, Ipswich, MA. USA) was used according to manufacturer’s guidelines to construct the cDNA libraries. The cDNA fragments were enriched by PCR amplification. The amplification product was purified using AMPure XP system. PCR products were purified (AMPure XP system) and library quality was assessed on the Agilent Bioanalyzer 2100 system. The 12 ovary tissues cDNA libraries were subjected to 2 × 150 bp paired-end sequencing using the Illumina NovaSeq 6000 platform (Illumina, San Diego, CA, USA).

For small RNA sequencing, the same sample was used to construct Illumina small RNA-seq (RNA sequencing) library by using the NEBNext®MultiplexSmall RNA Library Prep Set for Illumina kit® (NEB) following the manufacturer’s recommendations. In brief, the 5′ SR and 3′ SR adaptor for Illumina was ligated to the small RNA, and first strand cDNA was synthesized. The product was then subjected for the second strand synthesis. The product with 3′ SR and 5′ SR was then PCR amplified. PAGE gel was used to recover the 140-160 bp target fragment, rubber cutting recycling as the pieces get small RNA libraries. At last, PCR products were purified (AMPure XP system) and library quality was assessed. The 12 ovary tissues cDNA libraries were subjected to 1 × 50 bp single-end sequencing using the Illumina NovaSeq 6000 platform (Illumina, San Diego, CA, USA).

### Quality control

Raw data (raw reads) of fastq format were firstly processed through self perl scripts. Clean data (clean reads) were obtained by removing reads containing adapter, ploy-N and low quality reads. Further, the reads of miRNAs were trimmed by removing the sequences smaller than 18 nt or longer than 30 nt. The content of Q20, Q30 and GC and sequence duplication level of the clean data were calculated. All the downstream analyses were based on clean data with high quality.

### Analysis of RNA-Seq data

The clean reads were aligned to the geese reference genome (Ans Cyg_PRJNA183603_v1.0) by Hisat2(v2.0.4) [56], the mapped reads of each sample were assembled by StringTie (v1.3.1) [57]. StringTie(v1.3.1) [57] was used to assess expression levels of mRNAs and lncRNAs by calculating FPKM [58] (Fragments Per Kilobase of transcript per Million fragments mapped). DESeq2 [59] was used for differentially expressed gene (DEG) analysis and Benjamini and Hochberg’s [60, 61] method for FDR (False Discovery Rate) correction. Differential expression of mRNAs and lncRNA were defined according to the following criteria: |log2(Fold Change)| ≥1 and adjusted *P*-value (FDR) < 0.05.

### Analysis of microRNA-Seq data

The Clean Reads were aligned with Silva database, GtRNAdb database, Rfam database, and Repbase database, respectively, to filter ribosomal RNA (rRNA), transfer RNA (tRNA), small nuclear RNA (snRNA), small nucleolar RNA (snoRNA), repeat sequences, and other ncRNA using Bowtie tools (v1.0.0) [62], then unannotated reads containing miRNA were acquired. Unannotated reads were aligned to the geese reference genome (Ans Cyg_PRJNA183603_v1.0) using Bowtie(v1.0.0) [62], to obtain the location information on the reference genome (Mapped Reads). The small RNA that matched to the reference genome was compared to the miRNAs from all animals in the miRBase(v22) database to identify known miRNA. Novel miRNA prediction was performed using miRDeep2 (v2.0.5) [63]. For both known miRNAs and novel miRNAs, the miRNA expression level was calculated and normalized by transcripts per million (TPM) [64]. DESeq2(v1.10.1) [59] was used for differentially expressed miRNA analysis and Benjamini and Hochberg’s [60, 61] method for FDR correction. Differential expression of miRNAs were defined according to the following criteria: |log2(Fold Change)| ≥ 0.58 and *P*-value<0.01.

### lncRNAs identification

Transcript class_codes with “i”, “x”, “u”, “o”, “e”, length ≥ 200 bp and exon number ≥ 2, were selected, FPKM≥0.1, CPC (Coding Potential Calculator) [65], CNCI (coding-noncoding-index) [66], CPAT (coding potential assessment tool) [67] and Pfam-scan [68] were used to distinguish mRNAs from lncRNAs.

### GO and KEGG enrichment analyses

Coding genes located within 100 kb upstream and downstream of lncRNA were regarded as the cis-target genes of the lncRNA. Pearson correlation coefficient method was used to analyze the correlation between lncRNA and mRNA and the genes with |r| > 0.95 and *P* < 0.01 were considered as trans-target gene of the lncRNA. According to the gene sequence information of known miRNAs and novel miRNAs, miRanda [69] and targetscan [70] software were used to predict miRNA target genes. ClusterProfiler(v3.10.1) [71] and KOBAS (v2.0) [72] was used for GO (Gene Ontology) and KEGG (Kyoko Encyclopedia of Genes and Genomes) [73, 74] pathway analyses of differentially expressed mRNA, lncRNA target genes and miRNA target genes. GO enrichment analysis includes biological process (BP), cellular component (CC), and molecular function (MF). GO terms or KEGG pathways with corrected *p*-value (*q*-value) < 0.05 were considered to be significantly enriched.

### Construction of lncRNA-miRNA-mRNA networks

In this study, lncRNA-miRNA-mRNA network was constructed according to the ceRNA method with the following step, (1) The correlation of differentially expressed miRNA and mRNA was used to predict the negative interaction of miRNA-mRNA. (2) The predicted miRNA binding sites for the differentially expressed lncRNAs were identified using the miRanda [69], to construct a differentially expressed lncRNA-miRNA negative regulatory network. (3) Baes on the predicted miRNA-mRNA and lncRNA-miRNA regulatory network, the lncRNA-miRNA-mRNA network (ceRNA network) was constructed using Cytoscape(v3.8.0) [75] software.

### Reverse transcription real-time quantitative PCR (RT-qPCR)

The remaining RNA from RNA-seq library was used for RT-PCR quantification. Based on the lncRNA–miRNA–mRNA correlation networks, specifically, several interaction nodes were validated by RT-qPCR, including 9 mRNA, 6 lncRNA and 7 miRNA (Table S1). For mRNA, lncRNA and miRNA, RNA was reverse transcribed using RevertAid Reverse Transcriptase (Thermo Fisher Scientific) following the manufacturer’s protocol. Quantitative RT-PCR (RT-qPCR) was performed using AceQ Universal SYBR qPCR Master Mix (Vazyme Biotech, Nanjing, China) and ABI StepOnePlus machine (ABI, Foster City, CA, USA). The PCR protocol was initiated at 95 °C for 5 min, followed by 40 cycles of the amplification program, with denaturation at 95 °C, 15 s, and annealing/ extension at 60 °C, 30 s. At the end of the last amplification cycle, melt curves were generated to confirm the specificity of the amplification reaction. Primers were designed using Primer Premier 5.0. All primer sequences, including selected genes, miRNAs and internal control genes (GAPDH and U6 snRNA), were listed in Table S9. Relative expression levels of genes and miRNAs were calculated by the2^-ΔΔCt^ [76] method.

### Dual-luciferase reporter assays

PsiCheck2-MSTRG.5970.28-wild type (wt) / mutated (mut) and PsiCheck2-ERBB4-wt/mut vectors were synthesized by He Fei Yuanen Biotechnology Company (Hefei, Anhui, China). 293 T cells were seeded in a 24-well plates (5 × 10^5^ cells/well). Cells were co-transfected with wt or mut reporter vector and gga-miR-145–5p mimics or NC mimic duplexes using Lip 3000 (Invitrogen). At 48 h after transfection, cell lysates were prepared and dual luciferase reporter assay kit (RG027, Beyotime Institute of Biotechnology) was used to measure luciferase activities following the manufacturers instructions. The relative luciferase activities were calculated by comparing the Firefly/Renilla luciferase activity ratio.

### Statistical analysis

Statistical analysis was performed using the software IBM SPSS Statistics version 22. The comparative analysis of two groups was performed using Student’s t-test, and multiple comparative analysis was performed with one-way ANOVA. GraphPad Prism 8 was applied for making graph. It was considered to be statistically significant when *P*-value < 0.05.

## Supplementary Information

**Additional file 1: Table S1-8.** Length information of miRNA.

**Additional file 2: Table S2-12.** Information of overlap miRNAs.

**Additional file 3: Table S3-6**. KEGG analysis of differentially expressed mRNAs in CP vs PP.

**Additional file 4: Table S4-6.** KEGG analysis of the targets for differentially expressed lncRNAs in CP vs PP.

**Additional file 5: Table S5-6.** KEGG analysis of the targets for differentially expressed miRNAs in CP vs PP.

**Additional file 6: Table S6-3.** lncRNA-miRNA-mRNA regulation networks in CP vs PP.

**Additional file 7: Table S7-4.** Analysis of differential lncRNAs for each egg-laying period.

**Additional file 8: Table S8-1.** Differential lncRNAs involved in the reproductive process of Yili goose.

**Additional file 9: Table S9.** Quantitative real-time PCR primer sequences used in this study.

## Data Availability

The raw sequences were deposited into Sequence Read Archive (SRA) database with the BioProject accession number PRJNA825140. (https://www.ncbi.nlm.nih.gov/bioproject/825140).

## Abbreviations

PP: Pre-laying period
LP: Laying period
CP: Ceased-laying period
LncRNA: Long non-coding RNA
miRNA: MicroRNA
DEG: Differentially expressed gene
*PDGFRB*: Platelet derived growth factor receptor beta
*ERBB4*: Erb-b2 receptor tyrosine kinase 4
*LHCGR*: Luteinizing hormone/choriogonadotropin receptor
RNA-seq: RNA sequencing
rRNA: ribosomal RNA
tRNA: Transfer RNA
snoRNA: Small nucleolar RNA
FPKM: Fragments per kilobase of transcript per million fragments mapped
FDR: False discovery rate
GO: Gene Ontology
KEGG: Kyoko Encyclopedia of Genes and Genomes
BP: Biological process
CC: Bellular component
MF: Molecular function
RT-qPCR: Reverse transcription real-time quantitative PCR
*PGR*: Progesterone receptor
*FDFT1*: Farnesyl-diphosphate farnesyltransferase 1
*ADRA2A*: Adrenoceptor alpha 2A
*CP*: Ceruloplasmin; *GPNMB* Glycoprotein nmb
*CXCR4*: C-X-C motif chemokine receptor 4
*HGF*: Hepatocyte growth factor
*PTGER3*: Prostaglandin E receptor 3
*CTSS*: Cathepsin S
*FAS*: Fas cell surface death receptor
*RIPK1*: Receptor interacting serine/threonine kinase 1
*FADD*: Fas associated via death domain
*TRADD*: TNFRSF1A associated via death domain
*TNFRSF1A*: TNF receptor superfamily member 1A
*EGFR*: Epidermal growth factor receptor
*GNAS*: GNAS complex locus
*THBS1*: Thrombospondin 1
*TNC*: Tenascin C
*LPAR4*: Lysophosphatidic acid receptor 4
*WDFY4*: WDFY family member 4
*NRG1*: Neuregulin 1
*KIT*: KIT proto-oncogene: receptor tyrosine kinase
*NYAP2*: Neuronal tyrosine-phosphorylated phosphoinositide-3-kinase adaptor 2
*MYL9*: Myosin light chain 9
